# Improving the Accuracy of Single-Nucleotide Variant Diagnosis Using On–Off Discriminating Primers

**DOI:** 10.3390/bios13030380

**Published:** 2023-03-13

**Authors:** Juny Shin, Cheulhee Jung

**Affiliations:** Department of Biotechnology, College of Life Sciences and Biotechnology, Korea University, Seoul 02841, Republic of Korea

**Keywords:** single-nucleotide variant, on–off discrimination, polymerase chain reaction, low-abundance mutation, liquid biopsy

## Abstract

Early detection of rare mutations through liquid biopsy can provide real-time information related to cancer diagnosis, prognosis, and treatment outcomes. Cell-free DNA samples used in liquid biopsies contain single-nucleotide variants (SNVs) with a variant allele frequency (VAF) of approximately ≤1%. Droplet digital polymerase chain reaction (ddPCR) is considered the gold standard of sequencing using liquid samples, generating amplicons from samples containing mutations with 0.001–0.005% VAF; however, it requires expensive equipment and time-consuming protocols. Therefore, various PCR methods for discriminating SNVs have been developed; nonetheless, non-specific amplification cannot be avoided even in the absence of mutations, which hampers the accurate diagnosis of SNVs. In this study, we introduce single-nucleotide variant on–off discrimination–PCR (Soo-PCR), a highly accurate and practical method that uses a 3′-end tailing primer for the on–off discrimination of low-abundance mutant-type targets, including SNVs. Soo-PCR minimizes the chance of incorrect judgments owing to its high discriminating power. Cancer markers, such as KRAS G12D, EGFR L858R, and EGFR T790M mutations, containing 0.1% VAF, were clearly detected in under 2 h with a high reliability comparable with that of ddPCR. This new method serves as a practical approach to accurately detect and evaluate low-abundance mutations in a user-friendly manner.

## 1. Introduction

Early detection of rare mutations in the blood plasma provides real-time information related to tumor progression, treatment effectiveness, and cancer metastasis risk, and it has been a long-term goal in medical research [1,2,3,4]. As cancer cells undergo frequent cell division, apoptosis, and necrosis regardless of their location in the body, the resulting genomic DNA (gDNA) fragments remain in patients’ blood plasma [5]. Thus, performing liquid biopsies using DNA isolated from plasma has facilitated early detection and assessment of rare mutations, thereby providing important information for cancer diagnosis, prognosis, and treatment [6,7,8].

Liquid biopsies enable the detection and assessment of slight variations in DNA sequences and are convenient, minimally invasive, and reproducible [7,9,10]. Nevertheless, mutations, including single-nucleotide substitutions, in cell-free DNA (cfDNA) have a variant allele frequency (VAF) of ≤1%. Hence, a method that can selectively detect and assess low-abundance mutant-type sequence fragments from the background of abundant wild-type (WT) sequences derived from normal cells of the entire body is needed [11,12,13]. Various new sequencing and selective polymerase chain reaction (PCR) techniques have been developed to improve assay sensitivity and specificity. Next generation sequencing (NGS) techniques such as cancer personalized profiling by deep sequencing [14], tagged amplicon deep sequencing [6,15], and whole genome sequencing [16] can detect mutations with 0.02–2% (percentage of the mutant-type (MT) fraction) VAF and help identify correlations between mutation types and disease status. However, these methods are time-consuming (providing results in days to weeks) and expensive [3,17,18]. Droplet digital PCR (ddPCR) yields robust results from samples containing mutations with 0.001–0.005% VAF [6,19,20,21,22,23,24]. However, it requires expensive devices for droplet formation and detection and is a time-consuming protocol, which limits its use in smaller laboratories. In terms of price and practicality, a conventional PCR was engineered to amplify mutation sequences with 0.01–3% VAF by revising primers and probes. Previous studies have developed 3′ mismatch primer strategies such as double-mismatch allele-specific quantitative PCR [25] and dual priming oligonucleotide-PCR with two priming sites by the insertion of polydeoxyinosine linker [26,27,28,29], and engineered probe strategies such as suppression quantitative PCR using PNA, LNA [30,31,32], and X-probes [33]. Nonetheless, such approaches cannot avoid the background signals even in the absence of mutations, which hampers accurate single-nucleotide variant (SNV) identification. Due to the low specificity, distinguishing the cycle threshold (Ct) value in the absence of mutation from that in the presence of a low amount of mutation is difficult; thus, false positive/false negative outcomes are highly likely to occur. They have advantages in terms of price and practicality, but unfortunately they have a huge disadvantage in terms of accuracy.

In this study, to address the background signal issue of the engineered PCR methods, we focused on and improved a conventional 3′ mismatch primer strategy. Existing methods were not able to maximize the discriminating power of Taq polymerase, resulting in signal amplification even when only the WT was present. However, we significantly improved Taq polymerase’s SNV discrimination ability by adding a tail to the 3′-end, preventing amplification when only the WT is present. We named this method a single-nucleotide variant on–off discrimination–PCR (Soo-PCR). This method reduces associated costs, time, and labor while maintaining strong discrimination ability; it is capable of distinguishing low-abundance MT targets containing SNVs from the abundant WT background. Low-abundance mutations with 0.1% VAF were easily detected through on–off discrimination using Soo-PCR by optimizing the length of the primer’s 3′-end non-complementary nucleotides (tailing structure), annealing the temperature, and screening various Taq DNA polymerases. We used Horizon’s cfDNA (Horizon Discovery, Cambridge, UK) derived from human cell lines, which are fragmented to an average size of 160 bp to closely resemble cfDNA extracted from human plasma [34,35]. Low-abundance SNV fractions of KRAS G12D, EGFR L858R, and EGFR T790M cancer markers were detected in under 2 h, and the results were validated by comparing the data with those obtained using ddPCR. This method combines the advantages of existing SNV-PCR methods, such as price and practicality, while maximizing specificity to eliminate the issue of background signal and solve the inherent accuracy problems of previous methods. Therefore, this simple method can serve as a practical tool to accurately detect and evaluate low-abundance mutations from cfDNA, indicating its potential for clinical applications.

## 2. Materials and Methods

### 2.1. Design and Synthesis of Oligonucleotides for Soo-PCR

Oligonucleotides were designed and commercially synthesized using the PrimerQuest™ Tool (Integrated DNA Technologies (IDT), Coralville, IA, USA) for each target (KRAS G12D (c.35G > A), EGFR L858R (c.2573T > G), and EGFR T790M) c.2369C > T)). For designing primers (excluding the 3′-end tailing structure), the parameters were as follows: amplicon sizes between 60 and 80 nt, primer melting temperatures between 60 and 65 °C, and primer sizes between 18 and 25 nt. For designing the TaqMan probe, the parameters were as follows: Affinity Plus Probes (IDT) comprising 4–6 LNAs, probe melting temperatures between 66 and 72 °C, and probe sizes between 10 and 15 nt. All oligonucleotides used in this study are listed in Supplementary Tables S1–S3.

### 2.2. Screening Various Taq DNA Polymerases for Soo-PCR Primers with a 3′-End Tailing Structure

Five different Taq DNA polymerases—AmpliTaq Gold™ 360 Master Mix (Applied Biosystems, Waltham, MA, USA), Hot Start Taq 2× Master Mix (New England Biolabs, Ipswich, MA, USA), EzWay Hot Taq PCR Master Mix (KOMA BIOTECH, Seoul, Korea), DreamTaq™ Hot Start PCR Master Mix (Thermo Scientific, Waltham, MA, USA), and GoTaq^®^ Green Master Mix (Promega, Madison, WI, USA)—were screened for each of the two Soo-PCR primers (one that was a perfect match with the template at the penultimate (–2) base and another that was a mismatch at the same base) with a 3′-end tailing structure (tailing length: 0, 1, 2, 3, or 4 nt). The experiments were performed in triplicate. A total of 1500 copies (5 ng) of 100% KRAS wild-type gDNA (HD710, Horizon Discovery, Waterbeach, UK) containing only WT allelic frequency (AF) of the KRAS G12D target was used as a template.

The following constituents were used for all PCRs, regardless of the Taq DNA polymerase used: 1 μL of DNA template (5 ng μL^−1^), 10 μL of nuclease-free water (Invitrogen, Waltham, MA, USA), 12.5 μL of Taq master mix (1× final concentration), 0.5 μL of TaqMan probe (5 μM, IDT), 0.5 μL of forward primer (10 μM, IDT), and 0.5 μL of reverse primer (10 μM, IDT). 

Based on the Taq DNA polymerase used, the PCR cycling conditions were as follows. AmpliTaq: 95 °C for 10 min, followed by 70 cycles at 95 °C for 30 s, 56 °C for 30 s, and 72 °C for 15 s, and then at 72 °C for 7 min. Hot Start Taq: 95 °C for 30 s, followed by 70 cycles at 95 °C for 30 s, 56 °C for 30 s, and 68 °C for 15 s, and then at 68 °C for 5 min. EzWay: 95 °C for 15 min, followed by 70 cycles at 94 °C for 30 s, 56 °C for 30 s, and 72 °C for 15 s, and then at 72 °C for 10 min. DreamTaq: 95 °C for 3 min, followed by 70 cycles at 95 °C for 30 s, 61 °C for 30 s, and 72 °C for 1 min, and then at 72 °C for 7 min. GoTaq: 95 °C for 2 min, followed by 70 cycles at 95 °C for 30 s, 56 °C for 30 s, and 72 °C for 15 s, and then at 72 °C for 5 min (Table 1). We optimized the annealing temperature and 3′-end tailing structure length of Soo-PCR primers for KRAS G12D, EGFR L858R, and EGFR T790M. PCR performance was assessed using Soo-PCR primers with different 3′-end tailing structure lengths (0, 1, 2, 3, or 4 nt) at various PCR annealing temperatures. Two types of Soo-PCR primers were used: a perfect match with the template at the penultimate base and a mismatch at the same base. A total of 1500 copies (5 ng) of 100% X WT gDNA (Horizon Discovery, X: KRAS (HD710)/EGFR (HD709)) containing only WT AF were used as a template. All PCR experiments were performed in triplicate, and the following constituents were used: 1 μL of DNA template (5 ng μL^−1^), 10 μL of nuclease-free water (Invitrogen), 12.5 μL of AmpliTaq Gold™ 360 master mix (1× final concentration, Applied Biosystems), 0.5 μL of TaqMan probe (5 μM, IDT), 0.5 μL of forward primer (10 μM, IDT), and 0.5 μL of reverse primer (10 μM, IDT).

The following PCR cycling conditions were used: 95 °C for 30 s, annealing temperature X for 30 s (X: 56 °C, 58 °C, and 60 °C for KRAS G12D and EGFR T790M; 58 °C, 60 °C, and 62 °C for EGFR L858R), and 72 °C for 15 s, followed by 72 °C for 7 min.

### 2.3. Detection of Low-Abundance SNVs (KRAS G12D, EGFR L858R, and EGFR T790M)

The performance of Soo-PCR in amplifying low-abundance SNVs was assessed by using Soo-PCR to detect KRAS G12D, EGFR L858R, or EGFR T790M from gDNA and cfDNA standards (Horizon Discovery). Samples consisted of 0%, 0.1%, 1%, and 5% VAFs (20 ng μL^−1^) for each SNV allele. WT and mutant gDNA standards were mixed to a total of approximately 15,000 copies (50 ng) to obtain samples with 0%, 0.1%, 1%, 10%, and 50% VAFs for each SNV allele.

All PCR experiments were performed in triplicate, and the following constituents were used: X μL of DNA template (15,000 copies/X μL), nuclease-free water (to a total volume of 25 μL, Invitrogen), 12.5 μL of AmpliTaq Gold 360 master mix (1× final concentration, Applied Biosystems), 0.5 μL of TaqMan probe (5 μM, IDT), 0.5 μL of forward primer (10 μM, IDT), and 0.5 μL of reverse primer (10 μM, IDT).

The following PCR cycling conditions were used: 95 °C for 10 min, followed by 70 cycles at 95 °C for 30 s, annealing temperature X for 30 s (X: 56 °C for KRAS G12D and EGFR T790M; 62 °C for EGFR L858R), and 72 °C for 15 s, followed by 72 °C for 7 min.

## 3. Results and Discussion

### 3.1. Schematic Overview of Soo-PCR

**Figure 1:**
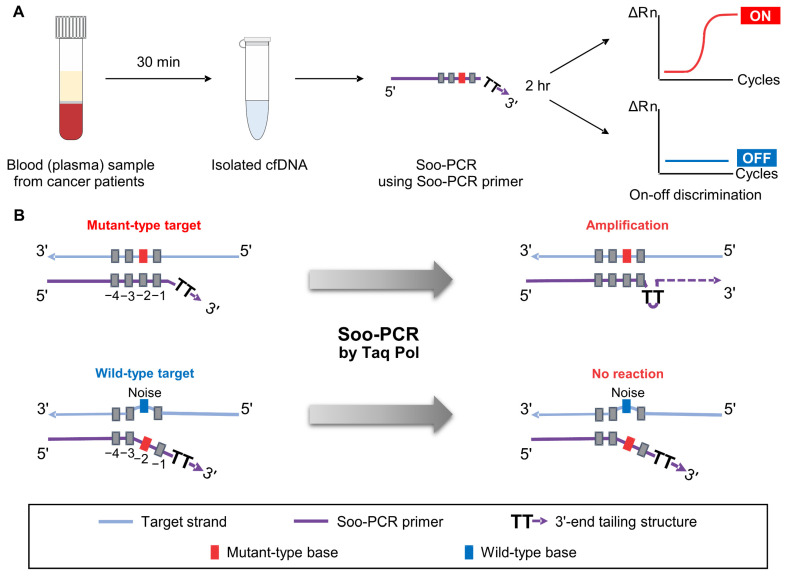
Schematic overview of Soo-PCR. (**A**) Liquid biopsy process with cell-free DNA isolated from blood sample (plasma) of a patient with cancer; Soo-PCR detects low-abundance cancer markers in an on–off discrimination format in under 2 h. (**B**) Soo-PCR scheme depicting on–off discrimination abilities based on the structural difference between primers with a 3′-end non-complementary nucleotide (tailing) structure of mutant-type sequence (**top**) and wild-type sequence (**bottom**).

The process of liquid biopsy used for early cancer diagnosis involves the purification of cfDNA from patient blood samples and the detection of specific mutations in the samples using NGS analysis or PCR techniques, such as ddPCR. The Soo-PCR method that we developed in this study by preparing specially designed PCR primers detected low-abundance cancer markers KRAS G12D, EGFR L858R, and EGFR T790M through on–off discrimination in under 2 h (Figure 1A).

It has been reported that Taq polymerase has a characteristic of ignoring short 3′ mismatches and proceeding with extension [36,37,38]. Therefore, there has been an issue of amplification even when there is a 3′ mismatch in previous strategies, and to address this, a double mismatch strategy was introduced to increase the length of the nucleotide that does not bind to the template when there is a mismatch [25]. The double-mismatch allele-specific qPCR (DMAS-qPCR) primer has a mismatch base to ensure that the SNV is located at the very end of the 3′. In addition, there is one non-complementary nucleotide to the target sequence at the fourth position (‘4’ base position) from the 3′ end, regardless of MT or WT. As a result, more bases exist in the form of ssDNA that do not bind to the template. However, because the non-mismatched portion in the 3′-end region is complementary to the template, it is not a condition where the fully single-stranded DNA form is maintained. Therefore, it can still partially bind to the template, resulting in some degree of amplification and consequently decreasing the discriminating power.

To address this issue, we focused on the tailing of the primer to create conditions where the 3′-end region can exist in an entirely ssDNA form. The Soo-PCR primer design is as follows: First, position the SNV at the penultimate position to create a 2 nt overhang structure due to the mismatch caused by the SNV. Second, add a non-complementary tail sequence to the 3′ end to make the 3′ end completely ssDNA. In this way, the primer forms a longer ssDNA 3′-end tailing structure when there is a mismatch, which cannot be ignored by Taq DNA polymerase, leading to significant suppression of amplification.

Therefore, it is important to choose which nucleotide to use as a tail to make the 3′ end as stable as possible in ssDNA form. The G and C nucleotides form a triple hydrogen bond, exhibiting a stronger hydrogen bond force than that between A and T [33,39,40], and induce non-specific primer extension. Furthermore, A and G nucleotides, which are purines containing aromatic rings, have a strong base stacking effect and weaken the overhang structure caused by the mismatch at the penultimate base. Hence, T nucleotides were selected to form the 3′-end tailing structures of the Soo-PCR primers.

### 3.2. Screening Taq DNA Polymerases for High Specificity

The Soo-PCR primers are designed to maximize the differential recognition of mismatched dsDNA by Taq polymerase. We anticipated that the structural stability of the primer/template hybrid would be greatly influenced by buffer composition and engineered active domains of the polymerase. Therefore, we conducted a screening process to optimize specificity conditions for on–off discrimination between the perfect-match target at the penultimate base (PMP template) and the mismatch target at the penultimate base (MP template) with various tail lengths of Soo-PCR primers using Taq DNA polymerases from multiple companies including AmpliTaq, Hot Start Taq, EzWay, DreamTaq, and GoTaq. This screening process also demonstrated experiments that clearly show specificity.

**Figure 2:**
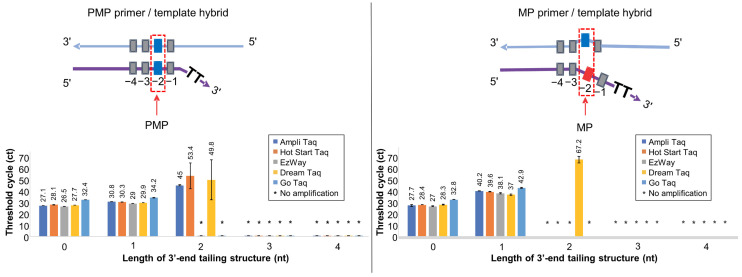
Screening various Taq DNA polymerases using different 3′-end tailing structures. Real-time PCR performance of Soo-PCR primers with a 3′-end tailing structure (tailing length: 0, 1, 2, 3, or 4 nt) for various Taq DNA polymerases (AmpliTaq Gold™ 360 master mix, Hot Start Taq 2× master mix, EzWay Hot Taq PCR master mix, DreamTaq™ Hot Start PCR master mix, and GoTaq^®^ Green master mix). Schematic depiction of the PMP primer/template hybrid (**top left**) and resulting Ct values (**bottom left**). Error bars represent the standard deviation from experiments performed in triplicate. Template: WT gDNA (Horizon Discovery) containing 1500 copies of WT AF for KRAS G12D.

High specificity, with regard to the Soo-PCR assay, refers to a stable on–off SNV discrimination performance. Here, on–off discrimination describes the ability to stably induce the amplification reaction in the PMP primer/template hybrid through the 3′-end tailing structure but not in the MP primer/template hybrid (Figure 2 top, 3′-end 2 nt (T) tailing has been included as an example). A stable amplification reaction refers to the convergence of triplicate results to the same value, and this was verified using the standard deviation of the Ct values. A sample was not considered to be amplified when only one value was obtained from the triplicate experiment. When one of the values was not obtained, the missing value was considered the Ct 70 (the last PCR cycle) for analysis. These considerations applied to all Ct value results of the real-time PCR assay. All Taq polymerases stably amplified PMP primer/template hybrids with the 0 nt and 1 nt tailing but not with the 3 nt and 4 nt tailing. Interestingly, a Ct value of 45 (SD = ±0.5) was obtained following amplification using AmpliTaq, a Ct value of 53.4 (SD = ±11.3) for Hot Start Taq, and a Ct value of 49.8 (SD = ±17.5) for DreamTaq when using the 3′-end 2 nt tailing. However, Hot Start Taq and DreamTaq displayed a highly unstable amplification reaction (SD > ±10). Conversely, AmpliTaq was associated with the most stable amplification reaction with the lowest Ct and SD values (Figure 2, bottom left). Additionally, all Taq DNA polymerases stably amplified MP primer/template hybrids with the 0 nt and 1 nt tailing but not with the 3 nt and 4 nt tailing. DreamTaq had a Ct value of 67.2 (SD = ±2.8) when the primer with the 3′-end 2 nt tailing was used. Unexpectedly, AmpliTaq and Hot Start Taq did not facilitate amplicon production (Figure 2). To comprehensively evaluate the discrimination ability of Soo-PCR primers for various Taq DNA polymerases, the difference in amplification efficiency between the PMP and MP primer/template hybrids was defined as ΔCtMP-PMP (MP Ct value—PMP Ct value). The Taq DNA polymerases did not amplify the PMP or MP primer/template hybrids with the 3′-end 3 nt and 4 nt tailing (Table 2). The five DNA polymerases also exhibited similar discrimination abilities when primers with the 3′-end 0 nt tailing (ΔCtMP-PMP = 0.3–0.6) and the 3′-end 1 nt tailing (ΔCtMP-PMP = 7.1–9.4) were used; their specificity for on–off discrimination was low. Unexpectedly, AmpliTaq and Hot Start Taq showed high specificities for the on–off discrimination (AmpliTaq, ΔCtMP-PMP = −45; Hot Start Taq, ΔCtMP-PMP = −53.4) when the primer with the 3′-end 2 nt tailing was used, whereas DreamTaq, which has a discrimination ability of ΔCtMP-PMP = 17.4 (Table 2), did not. AmpliTaq had a significantly higher ΔCtMP-PMP than Hot Start Taq and was associated with a more stable amplification of the PMP primer/template hybrid structure (SD = ±0.5). Hence, AmpliTaq was selected as the optimal polymerase for the on–off discrimination using the Soo-PCR assay (Figure 2 and Table 2). The polymerases used here are primarily based on Taq polymerase, but some may have been engineered, and the composition of the buffer used may vary slightly between each polymerase, resulting in different discriminating abilities. Therefore, it is possible to further improve discriminating ability by engineering polymerases or changing buffer composition.

Through Taq polymerase screening experiments, we were able to identify polymerase types and tailing lengths that can distinguish SNVs as on–off. Existing PCR methods that distinguish SNVs have low discriminating power, making it challenging to diagnose mutations accurately in extremely low amounts. In this regard, Soo-PCR, which demonstrates extremely high specificity through the tailing effect, is significant in the field of precision diagnosis that requires high accuracy.

### 3.3. Optimization of 3′-End Tailing Length of Soo-PCR Primer for Major Cancer Markers

**Figure 3:**
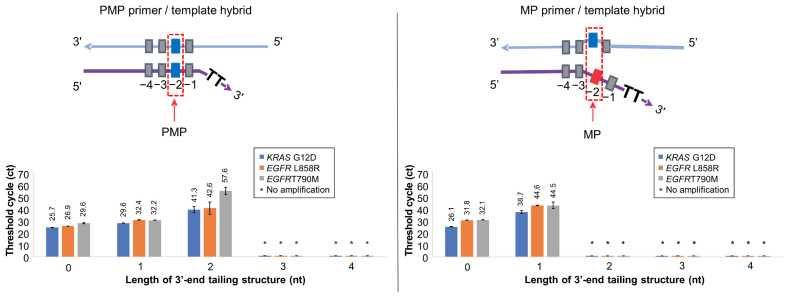
Optimizing the 3′-end tailing structure length of Soo-PCR primers for on–off discrimination of major cancer markers. Real-time PCR performance of Soo-PCR primers with a 3′-end tailing structure (tailing length: 0, 1, 2, 3, or 4 nt) on the PMP and MP primer/template hybrid of KRAS G12D (c.35G > A), EGFR L858R (c.2573T > G), and EGFR T790M (c.2369C > T). Schematic depiction of the PMP primer/template hybrid (**top left**) and the resulting Ct values (**bottom left**). Schematic depiction of the MP primer/template hybrid (**top right**) and the resulting Ct values (**bottom right**). Error bars represent the standard deviation from experiments performed in triplicate. Template: WT gDNA (Horizon Discovery) containing 1500 copies of WT AF KRAS G12D (c.35G > A), EGFR L858R (c.2573T > G), and EGFR T790M (c.2369C > T).

To evaluate the potential for cancer diagnosis with Soo-PCR, we selected KRAS G12D (c.35G > A), EGFR L858R (c.2573T > G), and EGFR T790M (c.2369C > T) among the cancer markers of major cancers such as stomach, colon, lung, thyroid, and breast cancer. For on–off discrimination of each target, we designed the target and Soo-PCR primers as follows. In both PMP and MP primer/template hybrid structures, the template for each target was prepared using wild-type genomic DNA (Horizon) containing only the WT allelic frequency (AF), with 1500 copies used. For each target, Soo-PCR primers were designed with ‘PMP’ and ‘MP’ primers that include tailing of various lengths (0–4 nucleotides). The ‘PMP’ primer was designed to have a perfect match with the template sequence at the penultimate (−2) base (Figure 3, top left), while the ‘MP’ primer was designed to have a mismatch with the template sequence at the penultimate (−2) base (Figure 3, top right).

In general PCR conditions, the annealing temperature is an essential factor that influences the specificity of PCR reactions. Therefore, in selecting the 3′-end tailing length of the Soo-PCR primer, we conducted experiments to optimize various annealing temperatures. Through these experiments, KRAS G12D and EGFR T790M were selected at 56 °C, and EGFR L858R was selected at 62 °C (Figure S1). The results shown in Figure 3 and Table 3 were obtained by performing annealing at a specific temperature optimized through annealing temperature optimization.

The PMP primer/template hybrid of all three targets showed no amplification when the 3′-end contained 3 nt and 4 nt tailing. However, stable amplification (Ct values, SD) was observed with 1 nt and 2 nt tailing of KRAS G12D: 0 nt (25.7, 0.1), 1 nt (29.6, 0.2), and 2 nt (41.3, 2.8); EGFR L858R: 0 nt (26.9, 0.1), 1 nt (32.4, 0), and 2 nt (42.6, 5.4); and EGFR T790M: 0 nt (29.6, 0.4), 1 nt (32.2, 0.1), and 2 nt (57.6, 3) (Figure 3). Interestingly, none of the three targets exhibited any amplification of the MP primer/template hybrid with 2 nt, 3 nt, or 4 nt tailing. However, there was stable amplification (Ct values, SD) with 0 nt and 1 nt tailing of KRAS G12D: 0 nt (26.1, 0.3) and 1 nt (38.7, 1.2); EGFR L858R: 0 nt (31.8, 0) and 1 nt (44.6, 0.3); and EGFR T790M: 0 nt (32.1, 0.1) and 1 nt (44.5, 2.8). The aforementioned ΔCtMP-PMP values were used to comprehensively assess Soo-PCR specificity. High specificity was observed for the on–off discrimination of KRAS G12D, EGFR L858R, and EGFR T790M using the 3′-end 2 nt tailing, which was similar to the primer structure obtained from Taq DNA polymerase screening (Table 3).

This observation is because the mismatch between the primer and the template affects both the stability and priming efficiency of the primer–template duplex. Structurally, a mismatch in the 3′-end structure is considered to interfere with the polymerase active site [36,37,38]. In the case of the 0 nt tail, the PMP primer has no overhang because it perfectly complements the template. On the other hand, the MP primer has a 2 nt overhang structure due to a mismatch caused by an SNV at the (−2) position relative to the template. Such a 2 nt overhang can be recognized by Taq polymerase with a high probability and amplified because they can partially anneal with the template. In the case of a 2 nt tail, PMP takes the form of a 2 nt tail, which can maintain its ssDNA form without fully annealing to the template. However, it seems highly likely that at least the 2 nt ssDNA can be easily recognized, as it is still amplified by Taq polymerase. On the other hand, in the case of MP, a 4 nt overhang structure is formed due to the consecutive existence of a 2 nt overhang and a 2 nt tail. It seems that Taq DNA polymerase does not recognize ssDNA of this length.

### 3.4. Validation of Soo-PCR Sensitivity and Specificity for Low-Abundance SNVs

**Figure 4:**
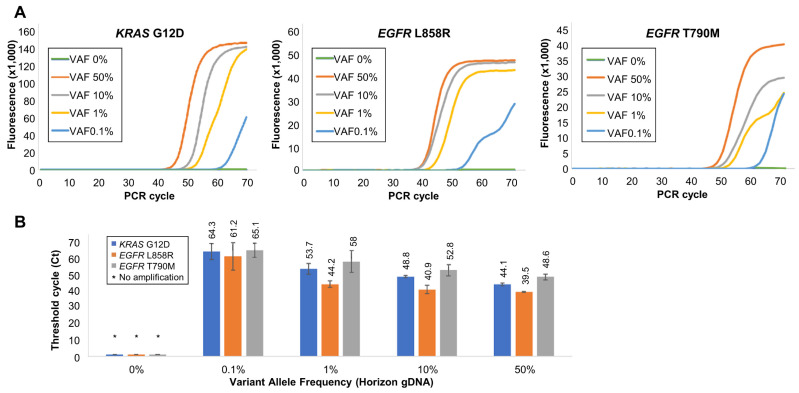
Validation of Soo-PCR assay sensitivity and specificity for each gDNA VAF. (**A**) Real-time PCR results of Soo-PCR for various mixtures of MT and WT gDNA for each target. (**B**) A summary of Ct values for the results observed in (**A**). Each sample mixture has a different VAF and a total of 15,000 gDNA copies (for example, 1% VAF = 150 MT copies and 14,850 WT copies). Error bars represent the standard deviation from experiments performed in triplicate. Target: KRAS G12D (c.35G > A), EGFR L858R (c.2573T > G), and EGFR T790M (c.2369C > T).

**Figure 5:**
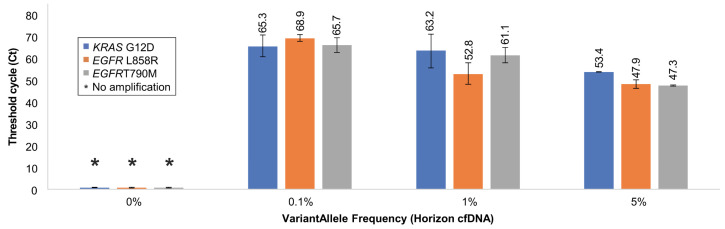
Validation of Soo-PCR assay sensitivity and specificity for each cfDNA VAF. Summary of Soo-PCR Ct values for various mixtures of MT and WT cfDNA for each target.

In liquid biopsy, cfDNA containing SNVs has a VAF of approximately 1% or lower [11,12,13]. Hence, highly sensitive and specific assays are required to detect these low-abundance mutant sequence fragments. Various VAF concentrations of gDNA (Horizon Discovery) and cfDNA (Horizon Discovery) were generated and amplified using the Soo-PCR assay to validate the sensitivity and specificity of the optimized Soo-PCR primers (Figure 4 and Figure 5).

One Soo-PCR primer with a complementary structure for MT sequence at the penultimate base was used, whereas a 3′-end 2 nt tailing structure was selected from the optimization experiment to determine the length of the 3′-end tailing structure of Soo-PCR primers for KRAS G12D (c.35G > A), EGFR L858R (c.2573T > G), and EGFR T790M (c.2369C > T). Validation was first performed with gDNA and then with the cfDNA. MT gDNA forming a PMP structure with the primer and WT gDNA forming a MP structure were mixed at various ratios for a total of 15,000 copies to produce different VAF concentrations. The VAF percentage was determined using the MT gDNA copy number. For instance, a 50% VAF indicates that 7500 copies of both MT and WT gDNA are present, and 10% VAF indicates that 1500 copies of MT gDNA and 13,500 copies of WT gDNA are present. A total copy number of 15,000 was selected to correspond with the maximum amount of cfDNA existing in blood plasma (liquid biopsy) according to previous reports [41,42,43].

The 0% VAF sample consisting of WT gDNA alone was not amplified for KRAS G12D, EGFR L858R, or EGFR T790M. Furthermore, the 0% VAF sample consisting of 135,000 copies of KRAS G12D WT gDNA was not amplified (Figure S2). This suggested that the amplification of samples with a VAF of 0.1% or higher was owing to the presence of MT gDNA. Furthermore, 50%, 10%, 1%, and 0.1% VAF samples based on the MT gDNA copy number were clearly amplified and the amplification efficiency increased as a function of MT gDNA percentage (Ct, SD) for KRAS G12D: 50% VAF (44.1, 0.8), 10% VAF (48.8, 0.7), 1% VAF (53.7, 3.3), and 0.1% VAF (64.3, 4.9); EGFR L858R: 50% VAF (39.5, 0.3), 10% VAF (40.9, 2.6), 1% VAF (44.2, 2.1), and 0.1% VAF (61.2, 8.5); and EGFR T790M: 50% VAF (48.6, 1.8), 10% VAF (52.8, 3.6), 1% VAF (58, 6.7), and 0.1% VAF (65.1, 4.3) (Figure 4A,B).

Based on the results obtained from gDNA (Horizon), we designed an experiment to validate the sensitivity and specificity of optimized Soo-PCR primers for cfDNA (Horizon) that closely resemble the form of clinical samples. Similar to the gDNA experiment, the total copy number of each sample was set to 15,000 and the VAF percentage was determined by the copy number of MT cfDNA; VAFs of 5%, 1%, 0.1%, and 0% were used. The results were quantified and validated using ddPCR, which is considered the gold standard of sequencing using liquid samples (Table 4). The on–off discrimination results were similar to those obtained in the gDNA type experiment. All 0% VAF samples consisting of WT cfDNA alone showed no amplification for each target. Conversely, 5–0.1% VAF samples showed clear amplification, and the amplification efficiency improved as a function of the MT cfDNA copy number (Ct, SD) value for KRAS G12D: 5% VAF (53.4, 0), 1% VAF (63.2, 7.7), and 0.1% VAF (65.3, 4.9); EGFR L858R: 5% VAF (47.9, 1.9), 1% VAF (52.8, 4.9), and 0.1% VAF (68.9, 1.6); and EGFR T790M: 5% VAF (47.3, 0.1), 1% VAF (61.1, 3.5), and 0.1% VAF (65.7, 3.3) (Figure 5 and Table 4). Therefore, Soo-PCR primers with a 3′-end 2 nt tailing structure distinguish gDNA and cfDNA samples containing mutations with a VAF of 0.1% through on–off discrimination with adequate sensitivity and specificity (Figure 5 and Table 4) and produce results comparable to those obtained using ddPCR.

Despite the accurate diagnosis that can be achieved through on–off detection, unfortunately, Soo-PCR faces difficulties in applying quantitative analysis accurately. Based on the data from Figure 5 and Table 4 of Soo-PCR, we drew standard curves for each gene (KRAS G12D, EGFR L858R, EGFR T790M). As a result, it was confirmed that the values of R^2^, which indicate quantitative measures, were not close to 1, with values of 0.8171, 0.9458, and 0.9085, respectively (Figure S3). This demonstrates that while it is not impossible to quantify, accurate quantification is difficult. Therefore, applying recovery analysis based on accurate quantification is also not easy. Although Soo-PCR has this limitation, it can be widely used in diagnostic fields where accuracy is important, as it has the unique advantage of accurately diagnosing small amounts of SNVs as on–off.

## 4. Conclusions

In summary, our study introduced a 3′-end-tailing-structure-mediated amplification reaction for on–off discrimination of SNVs present at low levels in plasma. The 3′-end tailing structure of the Soo-PCR primers can be easily designed owing to their practicality and simplicity. The unique 3′-end tailing structure of the Soo-PCR primers selectively induced Taq DNA polymerase-mediated amplification according to the presence of mismatched base pairs in the primer/template duplex. In existing strategies for detecting SNVs, such as 3′ mismatch primer and engineered probe methods, amplification occurs even when only WT is present, making it difficult to distinguish small amounts of MT due to the background signal. In contrast, Soo-PCR offers superior sensitivity and specificity, facilitating on–off discrimination against three major cancer markers (KRAS G12D, EGFR L858R, and EGFR T790M) present in low abundance (0.1% VAF, approximately 17 copies) in cfDNA samples closely resembling clinical samples in under 2 h. These results were comparable with those from ddPCR, which is recognized as the gold standard of sequencing using liquid samples. Furthermore, a 3′-end-tailing-structure-dependent amplification reaction can reduce cost by simplifying the primer design and only requires regular real-time PCR equipment. As it does not produce any background signals resulting in on–off discrimination, this new technique can serve as a practical tool to accurately detect and analyze low-abundance mutations in the cfDNA from plasma samples and has strong clinical application potential. Moreover, Soo-PCR is user-friendly and can be used as an endpoint diagnosis without requiring a qPCR device, thanks to its on–off system. Therefore, it can be combined with various nano-based diagnostic platforms, leading to potential synergistic effects [44,45].

## Figures and Tables

**Table 1:** PCR cycling conditions for Taq DNA polymerase screening.

Taq DNA Polymerase	PCR Cycling Conditions (°C/Times)				
	Initial Denaturation	70 Cycles			Final Extension
		Denaturation	Annealing	Extension	
Ampli Taq	95/10 min	95/30 s	56/30 s	72/15 s	72/7 min
Hot Start Taq	95/30 s	95/30 s	56/30 s	68/15 s	68/5 min
EzWay	95/15 min	94/30 s	56/30 s	72/15 s	72/10 min
Dream Taq	95/3 min	95/30 s	61/30 s	72/1 min	72/7 min
Go Taq	95/2 min	95/30 s	56/30 s	72/15 s	72/5 min

**Table 2:** ΔCtMP-PMP values for various Taq polymerases in Soo-PCR. ΔCtMP-PMP: Ct of MP primer/template hybrid–CT of PMP primer/template hybrid.

Taq DNA Polymerase	ΔCt_MP-PMP_				
	Length of 3′-End Tailing Structure (nt)				
	0	1	2	3	4
Ampli Taq	0.6	9.4	−45 **	*	*
Hot Start Taq	0.3	9.3	−53.4 **	*	*
EzWay	0.5	9.1	*	*	*
DreamTaq	0.6	7.1	17.4	*	*
GoTaq	0.4	8.7	*	*	*

* On-off discrimination ** No amplification.

**Table 3:** ΔCtMP-PMP values for the three cancer markers obtained using Soo-PCR.

Target	ΔCt_MP-PMP_				
	Length of 3′-End Tailing Structure (nt)				
	0	1	2	3	4
*KRAS* G12D	0.4	9.1	−41.3 **	*	*
*EGFR* L858R	4.9	12.2	−42.6 **	*	*
*EGFR* T790M	2.5	12.3	−57.6 **	*	*

* On-off discrimination ** No amplification.

**Table 4:** Summary of the comparative analysis between Soo-PCR and ddPCR quantitative results obtained for cfDNA samples in Figure 5. Each sample has a different VAF and a total of 15,000 cfDNA copies. Error bars represent the standard deviation from experiments performed in triplicate. Target: KRAS G12D (c.35G > A), EGFR L858R (c.2573T > G), and EGFR T790M (c.2369C > T).

Horizon cfDNA (HD780)		Droplet Digital PCR					Soo-PCR	
Gene	Variant	Copy Number			Expected AF (%)	Actual AF (%)	On/Off	Ct Value
		WT	MT	Total				
100% Wild Type								
*KRAS* G12D		14,995	5	15,000	0.00	0.02	Off	No amplification
*EGFR* L858R		15,000	0	15,000	0.00	0.00	Off	No amplification
*EGFR* T790M		14,993	7	15,000	0.00	0.05	Off	No amplification
0.1% Allele Frequency								
*KRAS* G12D		14,980	20	15,000	0.13	0.13	On	65.3
*EGFR* L858R		14,983	17	15,000	0.10	0.11	On	68.9
*EGFR* T790M		14,980	20	15,000	0.10	0.12	On	65.7
1% Allele Frequency								
*KRAS* G12D		14,822	178	15,000	1.30	1.17	On	63.2
*EGFR* L858R		14,870	130	15,000	1.00	0.87	On	52.8
*EGFR* T790M		14,878	122	15,000	1.00	0.81	On	61.1
5% Allele Frequency								
*KRAS* G12D		14,094	906	15,000	6.30	6.03	On	53.4
*EGFR* L858R		14,337	663	15,000	5.00	4.43	On	47.9
*EGFR* T790M		14,364	636	15,000	5.00	4.24	On	47.3

## Data Availability

Not applicable.

## Supplementary Materials

The following supporting information can be downloaded at: https://www.mdpi.com/article/10.3390/bios13030380/s1, Supplementary Table S1: Summary of oligonucleotides designed for KRAS G12D; Supplementary Table S2: Summary of oligonucleotides designed for EGFR L858R; Supplementary Table S3: Summary of oligonucleotides designed for EGFR T790M; Supplementary Figure S1: Optimization of Soo-PCR primer annealing temperatures for high specificity using various 3′-end tailing structure lengths; Supplementary Figure S2: Validation of Soo-PCR assay sensitivity and specificity for various combinations of WT gDNA and MT gDNA for KRAS G12D using polyacrylamide gel electrophoresis (PAGE). Supplementary Figure S3: Standard curves (including values of R^2^) of each gene for Figure 5 and Table 4.
