# Supplementary material for: Improving the Accuracy of Single-Nucleotide Variant Diagnosis Using On–Off Discriminating Primers
Source: Biosensors (Basel). 2023 Mar 13;13(3):380. doi: 10.3390/bios13030380 (PMC10046569; doi:10.3390/bios13030380)
Supplement: Supplementary file 1 [file biosensors-13-00380-s001.zip › biosensors-2232776-supplementary.pdf]

Supporting Information for

Improving the accuracy of single-nucleotide variant diagnosis using on-off discriminating primers

Juny Shin, Cheulhee Jung*

*Corresponding Author: Cheulhee Jung

Email: damo363@korea.ac.kr

This file includes:

Supplementary Figure S1: Optimization of Soo-PCR primer annealing temperatures for high specificity using various 3′-end tailing structure lengths
Supplementary Figure S2: Validation of Soo-PCR assay sensitivity and specificity for various combinations of WT gDNA and MT gDNA for KRAS G12D using polyacrylamide gel electrophoresis (PAGE).
Supplementary Figure S3: Standard curves (including values of R^2) of each gene for Figure 5 and Table 4.
Supplementary Table S1: Summary of oligonucleotides designed for KRAS G12D
Supplementary Table S2: Summary of oligonucleotides designed for EGFR L858R
Supplementary Table S3: Summary of oligonucleotides designed for EGFR T790M

A

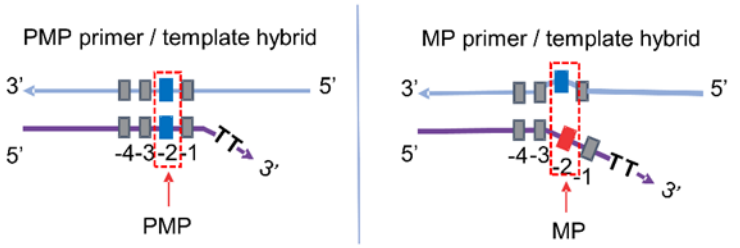

B

KRAS G12D					
Annealing temperature (°C)	ΔCt MP – PMP				
	Length of 3'-end tailing structure (nt)				
	0	1	2	3	4
56	0.4	9.1	-41.3 **	*	*
58	0.8	10.8	-50.6 **	*	*
60	1.9	10.7	-54.4 **	*	*

C

EGFR L858R					
Annealing temperature (°C)	ΔCt MP – PMP				
	Length of 3'-end tailing structure (nt)				
	0	1	2	3	4
58	1.9	8.5	29.2	*	*
60	2.8	10.3	29.9	*	*
62	4.9	12.2	-42.6 **	*	*

D

EGFR T790M					
Annealing temperature (°C)	ΔCt MP – PMP				
	Length of 3'-end tailing structure (nt)				
	0	1	2	3	4
56	2.5	12.3	-57.6 **	*	*
58	3.7	16	-60.5 **	*	*
60	3.7	18.8	-63.3 **	*	*

* No amplification ** On-off discrimination

Figure S1. Optimization of Soo-PCR primer annealing temperatures for high specificity using various 3'-end tailing structure lengths. (A) Schematic illustration of the PMP primer/template hybrid (left) and the MP primer/template hybrid (right) containing Soo-PCR primers with a 3'-end tailing structure. (B) Real-time PCR performance (ΔCtMP-PMP) of Soo-PCR primers with a 3'-end tailing structure for PMP and MP primer/template containing KRAS G12D (c.35G>A) at various annealing temperatures. (C) Real-time PCR performance (ΔCtMP-PMP) of Soo-PCR primers with a 3'-end tailing structure for PMP and MP primer/template containing EGFR L858R (c.2573T>G) at various annealing temperatures. (D) Real-time PCR performance (ΔCtMP-PMP) of Soo-PCR primers with a 3'-end tailing structure for PMP and MP primer/template containing EGFR T790M (c.2369C>T) at various annealing temperatures. PMP: perfect-match at the penultimate (-2) base, MP: mismatch at the penultimate (-2) base; Ct, threshold cycle; template, 1,500 copies of wild-type genomic DNA (Horizon) containing only WT allelic frequency (AF) for each target; ΔCtMP-PMP, Ct of MP primer/template hybrid – Ct of PMP primer/template hybrid.

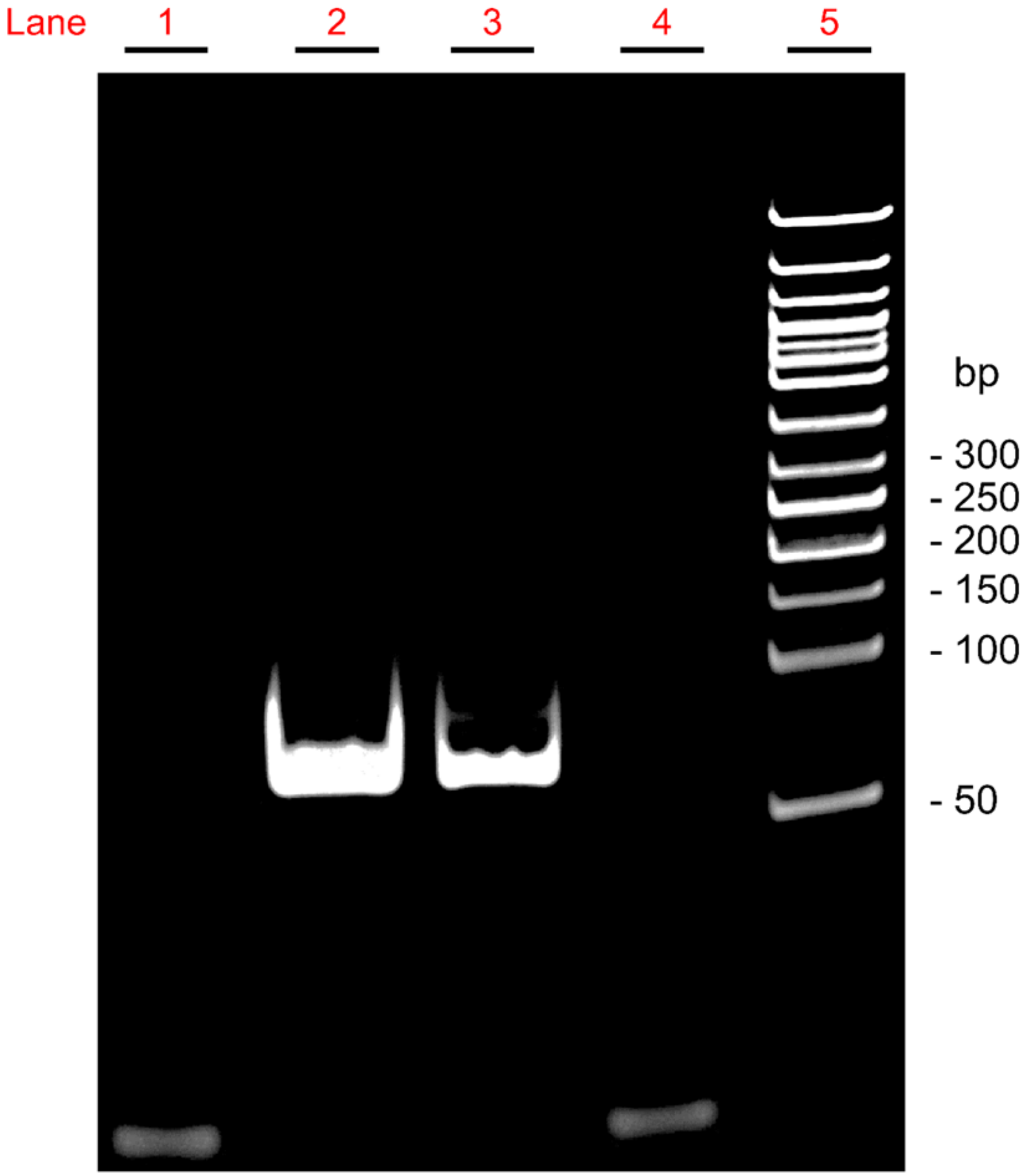

Figure S2. Validation of Soo-PCR assay sensitivity and specificity for various combinations of WT gDNA and MT gDNA for KRAS G12D using polyacrylamide gel electrophoresis (PAGE). PAGE result of the Soo-PCR assay products. Each Soo-PCR assay used a combination of WT and MT gDNA with a total of 135,000 copies. The PCR product size was 65 bp. Lane 1, 135,000 copies of WT gDNA; lane 2, 7,500 copies of MT and 127,500 copies WT of gDNA; lane 3, 750 copies of MT and 135,750 copies of WT gDNA; lane 4, 0 copies of MT and 0 copies of WT gDNA (control); lane 5, 50 bp DNA Ladder (New England Biolabs, Ipswich, MA, USA). All PCR reactions (lanes 1–4) had the same conditions except for the template.

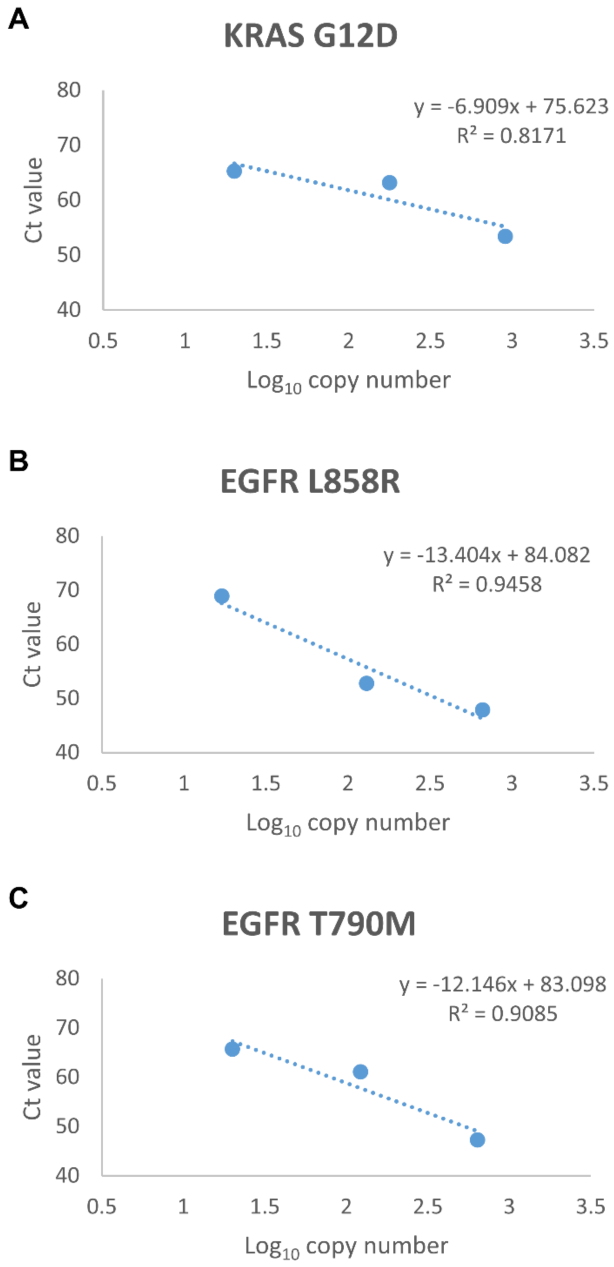

Figure S3. Standard curves (including values of R^2) of each gene for Figure 5 and Table 4. (A) Standard curve for KRAS G12D. (B) Standard curve for EGFR L858R. (C) Standard curve for EGFR T790M.

Table S1. Summary of oligonucleotides designed for KRAS G12D

Oligo Name		Sequence* (5'-3')	Length (nt)	Purification
Ultramer				
G12D_3′P-WT_Ultramer_v1		AAG GCC TGC TGA AAA TGA CTG AAT ATA AAC TTG TGG TAG TTG GAG CTG GTG GCG TAG GCA AGA GTG CCT TGA CGA TAC AGC TAA TTC AGA ATC ATT TTG TGG ACG AAT ATG ATC CAA CAA TAG AGG TAA ATC TTG TTT TAA TAT GCA TAT TAC TGG TGC AGG ACC ATT CTT TGA TAC AGA TAA AGG TTT CTC TGA /3Phos/	195	PAGE
G12D_3′P-MT_Ultramer_v1		AAG GCC TGC TGA AAA TGA CTG AAT ATA AAC TTG TGG TAG TTG GAG CTG ATG GCG TAG GCA AGA GTG CCT TGA CGA TAC AGC TAA TTC AGA ATC ATT TTG TGG ACG AAT ATG ATC CAA CAA TAG AGG TAA ATC TTG TTT TAA TAT GCA TAT TAC TGG TGC AGG ACC ATT CTT TGA TAC AGA TAA AGG TTT CTC TGA /3Phos/	195	PAGE
Forward Primer (FP)	Length of 3'-end tailing structure (nt)			
KRAS G12D_MT_T0	0	TTG TGG TAG TTG GAG CTG **A**T	20	HPLC
KRAS G12D_WT_T0	0	TTG TGG TAG TTG GAG CTG **G**T	20	HPLC
KRAS G12D_MT_T1	1	TTG TGG TAG TTG GAG CTG **A**T <u>T</u>	21	HPLC
KRAS G12D_WT_T1	1	TTG TGG TAG TTG GAG CTG **G**T <u>T</u>	21	HPLC
KRAS G12D_MT_T2	2	TTG TGG TAG TTG GAG CTG **A**T <u>TT</u>	22	HPLC
KRAS G12D_WT_T2	2	TTG TGG TAG TTG GAG CTG **G**T <u>TT</u>	22	HPLC
KRAS G12D_MT_T3	3	TTG TGG TAG TTG GAG CTG **A**T <u>TTT</u>	23	HPLC
KRAS G12D_WT_T3	3	TTG TGG TAG TTG GAG CTG **G**T <u>TTT</u>	23	HPLC
KRAS G12D_MT_T4	4	TTG TGG TAG TTG GAG CTG **A**T <u>TTTT</u>	24	HPLC

KRAS G12D_WT_T4	4	TTG TGG TAG TTG GAG CTG **G**T TTTT	24	HPLC
Reverse Primer (RP)				
KRAS G12D_RP		ATG ATT CTG AAT TAG CTG TAT CGT C	25	HPLC
TaqMan Probe				
KRAS G12D_TaqMan Probe		/56-FAM/ CA+C T+CT +T+G+C +CT /3IABkFQ/	11	HPLC

* Bold font: single nucleotide variant (SNV); Underline: 3'-end tailing structure
PAGE, polyacrylamide gel electrophoresis; HPLC, high-performance liquid chromatography

Table S2. Summary of oligonucleotides designed for EGFR L858R

Oligo Name		Sequence* (5'-3')	Length (nt)	Purification
Forward Primer (FP)	Length of 3'-end tailing structure (nt)			
EGFR L858R_WT_T0	0	CA AGA TCA CAG ATT TTG GGC **T**G	22	HPLC
EGFR L858R_MT_T0	0	CA AGA TCA CAG ATT TTG GGC **G**G	22	HPLC
EGFR L858R_WT_T1	1	CA AGA TCA CAG ATT TTG GGC **T**G T	23	HPLC
EGFR L858R_MT_T1	1	CA AGA TCA CAG ATT TTG GGC **G**G T	23	HPLC
EGFR L858R_WT_T2	2	CA AGA TCA CAG ATT TTG GGC **T**G TT	24	HPLC
EGFR L858R_MT_T2	2	CA AGA TCA CAG ATT TTG GGC **G**G TT	24	HPLC
EGFR L858R_WT_T3	3	CA AGA TCA CAG ATT TTG GGC **T**G TTT	25	HPLC
EGFR L858R_MT_T3	3	CA AGA TCA CAG ATT TTG GGC **G**G TTT	25	HPLC
EGFR L858R_WT_T4	4	CA AGA TCA CAG ATT TTG GGC **T**G TTTT	26	HPLC
EGFR L858R_MT_T4	4	CA AGA TCA CAG ATT TTG GGC **G**G TTTT	26	HPLC
Reverse Primer (RP)				

EGFR L858R_RP		GCC TCC TTC TGC ATG GTA TT	20	HPLC
TaqMan Probe				
EGFR L858R_TaqMan Probe		/56-FAM/ GCT +GG+G T+GC +GG+A AG /3IABkFQ/	14	HPLC

* Bold font: single nucleotide variant (SNV); Underline: 3′-end tailing structure
HPLC, high-performance liquid chromatography

Table S3. Summary of oligonucleotides designed for EGFR T790M

Oligo Name		Sequence* (5'-3')	Length (nt)	Purification
Forward Primer (FP)	Length of 3'-end tailing structure (nt)			
EGFR T790M_WT_T0	0	ACC GTG CAG CTC ATC A**C**G	18	HPLC
EGFR T790M_MT_T0	0	ACC GTG CAG CTC ATC A**T**G	18	HPLC
EGFR T790M_WT_T1	1	ACC GTG CAG CTC ATC A**C**G T	19	HPLC
EGFR T790M_MT_T1	1	ACC GTG CAG CTC ATC A**T**G T	19	HPLC
EGFR T790M_WT_T2	2	ACC GTG CAG CTC ATC A**C**G TT	20	HPLC
EGFR T790M_MT_T2	2	ACC GTG CAG CTC ATC A**T**G TT	20	HPLC
EGFR T790M_WT_T3	3	ACC GTG CAG CTC ATC A**C**G TTT	21	HPLC
EGFR T790M_MT_T3	3	ACC GTG CAG CTC ATC A**T**G TTT	21	HPLC
EGFR T790M_WT_T4	4	ACC GTG CAG CTC ATC A**C**G TTTT	22	HPLC
EGFR T790M_MT_T4	4	ACC GTG CAG CTC ATC A**T**G TTTT	22	HPLC
Reverse Primer (RP)				
EGFR T790M_RP		C TTT GTG TTC CCG GAC ATA GT	21	HPLC

TaqMan Probe				
EGFR T790M_TaqMan Probe		/56-FAM/ C A+TG +CC+C T+TC GGC T /3IABkFQ/	14	HPLC

* Bold font: single nucleotide variant (SNV); Underline: 3'-end tailing structure
HPLC, high-performance liquid chromatography
